# SS-Wrapper: a package of wrapper applications for similarity searches on Linux clusters

**DOI:** 10.1186/1471-2105-5-171

**Published:** 2004-10-28

**Authors:** Chunlin Wang, Elliot J Lefkowitz

**Affiliations:** 1 Department of Microbiology, University of Alabama at Birmingham, Birmingham, Alabama 35294-2170, USA

## Abstract

**Background:**

Large-scale sequence comparison is a powerful tool for biological inference in modern molecular biology. Comparing new sequences to those in annotated databases is a useful source of functional and structural information about these sequences. Using software such as the basic local alignment search tool (BLAST) or HMMPFAM to identify statistically significant matches between newly sequenced segments of genetic material and those in databases is an important task for most molecular biologists. Searching algorithms are intrinsically slow and data-intensive, especially in light of the rapid growth of biological sequence databases due to the emergence of high throughput DNA sequencing techniques. Thus, traditional bioinformatics tools are impractical on PCs and even on dedicated UNIX servers. To take advantage of larger databases and more reliable methods, high performance computation becomes necessary.

**Results:**

We describe the implementation of SS-Wrapper (Similarity Search Wrapper), a package of wrapper applications that can parallelize similarity search applications on a Linux cluster. Our wrapper utilizes a query segmentation-search (QS-search) approach to parallelize sequence database search applications. It takes into consideration load balancing between each node on the cluster to maximize resource usage. QS-search is designed to wrap many different search tools, such as BLAST and HMMPFAM using the same interface. This implementation does not alter the original program, so newly obtained programs and program updates should be accommodated easily. Benchmark experiments using QS-search to optimize BLAST and HMMPFAM showed that QS-search accelerated the performance of these programs almost linearly in proportion to the number of CPUs used. We have also implemented a wrapper that utilizes a database segmentation approach (DS-BLAST) that provides a complementary solution for BLAST searches when the database is too large to fit into the memory of a single node.

**Conclusions:**

Used together, QS-search and DS-BLAST provide a flexible solution to adapt sequential similarity searching applications in high performance computing environments. Their ease of use and their ability to wrap a variety of database search programs provide an analytical architecture to assist both the seasoned bioinformaticist and the wet-bench biologist.

## Background

Evolution can be measured and studied on a number of different scales, one of which is through the determination and comparison of genetic sequence information. Current-day gene sequences in living organisms have arisen through modifications of an array of ancestral sequences. Duplication with modification is the central paradigm of protein evolution, wherein new proteins and/or new biological functions are fashioned from earlier ones. [1]. To detect these functionally (and structurally) related proteins based upon similarity between their primary nucleic acid or amino acid sequences, a variety of sequence comparison algorithms have been developed. When a new gene is cloned and sequenced, it is now standard practice to use these algorithms to search for similarities between the translated nucleic acid sequence and a protein sequence database such as the NCBI non-redundant protein database (nr) [2]. Sequence similarity that implies similar structure and therefore similar protein or enzymatic function is not definitive proof of such function; however, the results of database sequence similarity searches provide a starting point for researchers attempting to ascertain the function of an unknown gene by supporting the intelligent design of subsequent laboratory experiments.

The basic local alignment search tool (BLAST) [3,4] is by far the most widely used pairwise-based sequence similarity comparison tool. It completes searches more swiftly than other tools, including FASTA, SSEARCH [5], and SCANPS [6]. BLAST uses an efficient, rapid algorithm to look for short segments or words of sequence similarity between two sequences that meet some predefined scoring threshold. After initially locating at least two of these words within a short distance of one another on a common diagonal, the algorithm uses them as "seeds" from which to extend the alignment to encompass longer regions of similarity, resulting in high scoring pairs (HSPs). The heuristic algorithm used by BLAST decreases search time dramatically in comparison to that of other search programs. However, the emergence of high-throughput DNA sequencing techniques has increased the size of sequence databases tremendously; thus conventional large-scale BLAST searching against the most commonly used databases has become infeasible on a PC or even a dedicated UNIX server. For this reason, new search strategies are needed.

While BLAST provides a balance between search sensitivity and speed, in many cases a researcher would like to detect more distant sequence similarities by employing search strategies that maximize the sensitivity of the search. A profile-based comparison which, for example, compares a sequence to a hidden Markov model (HMM) representing an empirically derived estimate of all possible evolutionary changes for a protein of a particular function, generally permits identification of a much higher proportion of distantly related sequences [7]. There are two major profile-based comparison tools. PSI-BLAST [3] compares sequences with a profile model constructed dynamically during the initial search phase of a traditional BLAST search, while HMMPFAM in the HMMer package from Sean Eddy at Washington University compares sequences with a well-curated database of HMM profiles as well as models constructed by users. Well-curated profile databases such as Pfam [8] are being developed through the combined efforts of bioinformaticists and molecular biologists. Profile-based comparison has become a reliable way to gather information for predicting structure and function of unknown genes, and tools from the HMMer package are becoming a key centerpiece in many bioinformatics pipelines. The tradeoff of using HMM-based searches for increased sensitivity is the intrinsically slow nature of the Viterbi [9] or forward algorithm used in the application.

Taking advantage of larger databases and more sensitive searching methods necessitates the use of high performance computing (HPC) platforms. Traditionally, HPC has been synonymous with high-priced vector or parallel supercomputers, but rapid advances in microprocessor and network bandwidth technologies are changing the definition [10]. Clusters of connected workstations utilizing commodity microprocessor systems provide enormous benefits in terms of cost and performance. Thus, cluster computing can meet the increased computational needs of resource- and data-intensive bioinformatics applications. HPC environments using workstations connected via high-speed networks are becoming more and more popular in the bioinformatics community [11-14]. Here we describe the SS-Wrapper package, which provides tools to adapt currently available similarity search applications onto HPC environments implemented through Linux clusters.

## Implementation

The objective of this study was to implement a generic wrapper application that could deploy similarity searching applications on a Linux cluster. Design criteria for the wrapper included the ability to deploy applications without the need to alter the original application and the ability to increase the speed of the underlying application in a linear manner dependent on the number of cluster nodes available. To meet these objectives, tools in the SS-Wrapper package were written in C/C++ using the message passing interface (MPI) [15]. Tools in the SS-Wrapper should work with few if any changes on any Linux cluster running MPI utilizing any hardware platform. In addition to the SS-Wrapper executables (which are compiled using the MPI C++ compiler), executables for the underlying similarity search applications (blastall, formatdb, and hmmpfam) appropriate for the hardware platform underlying the cluster will be needed. These can be obtained from NCBI and Washington University in St. Louis. SS-Wrapper is available without charge under the Artistic License described in the Open Source Initiative [16]. The source code can be downloaded via ftp [17].

Using multiple processors is practical only when the computational task is too large to complete on a single processor in a reasonable amount of time. In the case of database searching, the database to be searched may be too large or the set of query sequences used in the search may be too large to accomplish the search on a workstation with a single or even double or quad processor architecture. Since the search must compare every query sequence to every database sequence, parallelizing the process can be accomplished by three different methods. The first method is to split the query sequence file into smaller subsets and apply each subset to one particular node of the cluster in a search against the entire database. The second method calls for the database to be split into a series of smaller files, one of which is distributed to each node of the cluster; then the entire file of query sequences is searched against each of the database segments. Finally, it is possible to use a combination of query sequence splitting and database splitting to accomplish the search.

Using the query splitting approach does not require any modification to the output generated by search programs such as BLAST. When utilizing the database splitting approach, however, a correction must be made to the reported E-value for any particular hit. Most similarity comparison tools provide a statistical measure (e.g., the E-value reported for BLAST and HMMPFAM) that gives an indication of the statistical significance of a match between the query sequence and a particular database hit. This statistical measure is generally influenced by the size of the search space (which includes the total length of the database) and therefore the E-value needs to be recalculated when only a portion of the database is being searched. An added complication is that some search tools require that the database be intact. For example, PSI-BLAST [3] is a variant of BLAST that constructs a sequence profile model based on hits from an initial round of BLAST searching. This profile is then used and refined in subsequent rounds of searching to increase the sensitivity of the overall search. Because PSI-BLAST depends on the integrity of the database to guarantee that the resulting profiles are representative of the entire search space, it is best to perform parallelization using the query splitting approach.

In contrast to database splitting, query splitting offers greater flexibility in that the query file segments can more easily be adaptively distributed to the nodes of the cluster according to the load on each particular node during the search process. As any one node becomes available, another segment of the query file can be distributed to that node during the search process, which improves performance. It is difficult to predict the workload on any one node before the search begins, and the time required to complete a program running on a cluster depends on the processor that finishes last. Adaptive distribution of the workload maximizes resource utilization. Optimizing resource utilization is dependent on finding a balance between having larger numbers of smaller tasks versus increased startup and communication overhead due to distribution of the required query and database files to each node. For this reason, for our query splitting wrapper (QS-search), we adopted a hybrid strategy wherein approximately 90% of the total workload is evenly divided and distributed to each node at the beginning of the search using a modified bucket algorithm (described below). Then the remaining 10% of the workload is divided and distributed to each node as it becomes available after completing its previous task. This strategy is accomplished using a master-slave model, where one node is set aside to act as the master, which is responsible for distributing the workload and supervising the slave nodes, which perform the computations.

In general, the query splitting approach seems to be superior to the database splitting approach due to higher performance and fewer post-search processing tasks. Database splitting does provide a distinct advantage when the database is too large to fit into the physical memory of a single node [11,14]. NCBI BLAST uses memory mapped file I/O for database access. BLAST runs fastest when it can cache the database in memory. When the database size exceeds that of the available memory, however, the database splitting approach can reduce the possibility of swapping the database from physical memory to disk swap space, which could significantly slow the search process. Therefore, we have also developed a wrapper to support database splitting (DS-BLAST). DS-BLAST is specific for BLAST because it is necessary to include code to recalculate E-values following the search.

As indicated above, a modified bucket algorithm is used to split up the query sequences for QS-search, and a similar algorithm is used to split up the database sequences for DS-BLAST. The modified bucket algorithm works as follows: First, the sequences are sorted according to length. In the first cycle, sequences are placed one at a time into each bucket in descending order of length. In the next two cycles, individual sequences continue to be placed into each bucket after first reversing the order of the buckets. Bucket order is reversed every two cycles and the process continues until all sequences have been placed into a bucket. At the end of this process, each bucket contains nearly the same number of sequences, and the total length of all sequences in any one bucket is also approximately the same as the total sequence length of any other bucket. In the end, therefore, the file of query sequences or the file of database sequences is evenly divided in both length and number.

**Figure 1:**
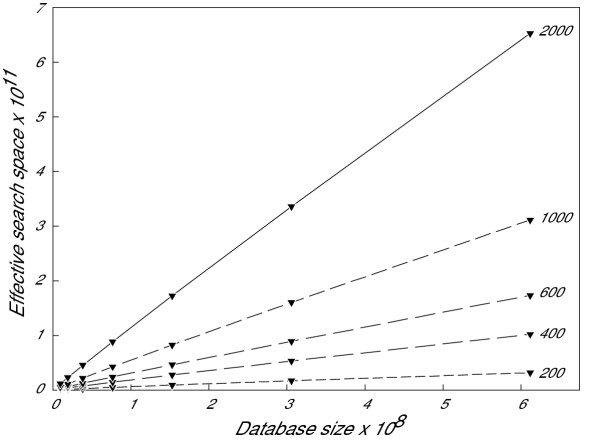
**Effective search space vs. database size and query sequence length. **Relationship between effective search space and database size with different query sequence lengths. The GenBank non-redundant protein database (nr) was split evenly according to the modified bucket algorithm in order to construct databases of a size 1/64, 1/32, 1/16, 1/8, 1/4, 1/2, or 1 of the entire nr database. Query sequences of varying lengths were randomly assembled using a Perl script. A BLAST search was then carried out for each query sequence against each database. The effective search space and database size was extracted from the BLAST results and plotted for each query sequence. The length of each query sequence is indicated next to the line which plots the relationship between effective search space and database size for that query.

The BLAST E-value is a function of the size of the effective search space, which is dependent on three factors: the number of sequences in the database, the total combined length of all sequences in the database, and the length of the query sequence [13,18]. Figure 1 shows that when splitting the database into N fragments of equal sequence number and length, the effective search space of each database fragment is approximately 1/N that of the intact entire database. Therefore, the E-value calculated for any particular hit of a query sequence to a database sequence will approximate a linear function dependent on the value of N. For that reason, at the beginning of the search, DS-BLAST lowers the user-provided E-value cut-off to account for the number of nodes used in the search. Following the search, DS-BLAST recalculates the effective search space and each resulting E-value by multiplying by the value of N.

## Results and discussion

### Usage

The QS-search executable (qssearch) provides the same interface for all search tools. The command line is as follows:

qssearch -c <command> -q <query> -d <database> -o <output> -l <local scratch> -x <database files>

DS-BLAST uses two executables: dsblast and dsformatdb. dsformatdb is responsible for splitting the database into fragments according to the modified bucket algorithm and then formats these fragments using the NCBI formatdb executable.

The command line for dsformatdb is as follows:

dsformatdb -n <number> -c <command> -d <database> -p <path>

The command line for dsblast is as follows:

dsblast -o <output> -c <command> -l <local scratch> -d <database> -q <query>

The command-line variables are as follows:

• -c command: normal command line used for the underlying application including all desired options

• -q query: query filename in fasta format

• -d database: database filename

• -o output: output filename

• -l local scratch: temporary directory on each node

• -x database files: a space-delimited list of the database file names generated by the search program's formatting utility (formatdb for BLAST)

• -n number: desired number of database fragments

• -p path: directory to store database fragments.

### Benchmarking

All benchmark experiments were performed on a Linux cluster in the Department of Engineering at the University of Alabama at Birmingham [19]. The cluster consists of one compile node and 64 compute nodes (IBM × 335s), as well as 2 storage servers (IBM × 345s). All machines have 2 × 2.4 GHz Xeon processors, 2 GB of RAM, an 18 GB SCSI hard drive, and are connected via Gigabit Ethernet to a Cisco 4006 switch. The NCBI non-redundant protein database (nr, 733 MB), downloaded from GenBank [2] in August, 2003, was used for testing both DS-BLAST and QS-search; it contained 1,508,485 sequences composed of 492,678,715 amino acids. Release 10.0 of the Pfam [8] database from Washington University (549 MB) was used in benchmarking HMMPFAM under QS-search; it contained 6190 profile models that, when combined, were 1,463,477 residues long. The same set of query sequences was used for all experiments. The query sequences represented all open reading frames of more than 30 amino acids from the genome of monkeypox virus strain WRAIR 7–61 (manuscript in preparation), and totaled 2068 sequences comprising 151,173 amino acids.

**Figure 2:**
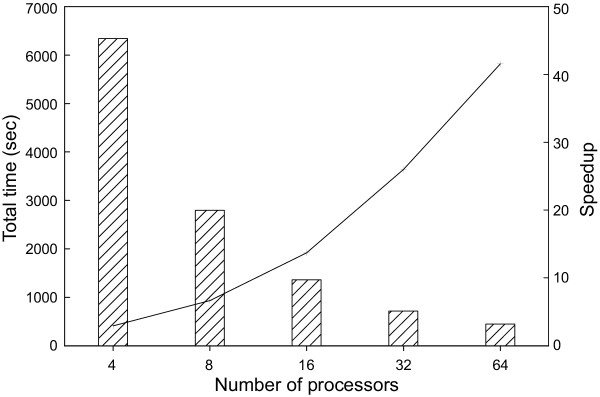
**BLAST performance of QS-search. **Performance of QS-search with NCBI BLAST when searching all reading frames (>30 amino acids) from monkeypox virus against the GenBank non-redundant protein database. Vertical bars represent total time used while the line indicates increase in speed corresponding to the number of processors used.

**Figure 3:**
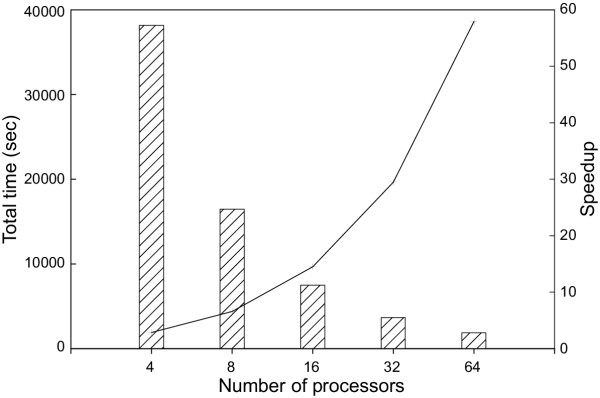
**HMMPFAM performance of QS-search. **Performance of QS-search for HMMPFAM when searching all reading frames (>30 amino acids) from monkeypox virus genome against Release 10.0 of the Pfam database. Vertical bars represent total time used while the line indicates increase in speed corresponding to the number of processors used.

Figure 2 demonstrates that QS-search provided a >40-fold acceleration for NCBI BLAST when using 64 processors, compared to the speed of 1 processor. Ideally, QS-search should provide an N-fold acceleration when using N processors, but this optimal result is rarely achievable. The major factor limiting the performance gain of QS-search is the time necessary to deliver the database to each node. As more nodes are employed, the portion of time spent searching decreases, but the communication overhead increases. When using QS-search with HMMPFAM, the search resulted in a 58-fold increase in processing speed when using 64 processors compared to a single processor (figure 3). QS-search therefore proved to be more efficient when running HMMPFAM in comparison to BLAST. Since the overall time required for the HMMPFAM search is much longer than that for BLAST, the portion of the search time devoted to communication overhead decreases thus increasing overall efficiency.

**Figure 4:**
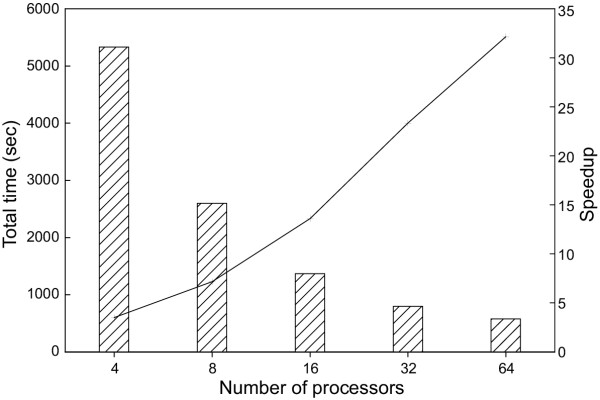
**Performance of DS-BLAST. **Performance of DS-BLAST when searching all reading frames (>30 amino acids) from monkeypox virus genome against the GenBank non-redundant protein database. Vertical bars represent total time used while the line indicates increase in speed corresponding to the number of processors used.

Figure 4 illustrates the performance of DS-BLAST. Since database preprocessing occurs before the search process, the times provided in figure 4 do not include this preprocessing time. As the same query, database and underlying application (NCBI-BLAST) were used in benchmarking DS-BLAST and QS-search, the results presented in figures 2 and 4 are directly comparable and indicate that QS-search appears to be more efficient than DS-BLAST. Two factors cause a reduction in performance of DS-BLAST. The first is the imbalance of the workload between nodes, and the second is the time necessary for the final merge phase of the output results from each node. When using QS-search, the workload is distributed dynamically during execution and therefore is well balanced between nodes. In contrast, when using DS-BLAST, the database is split into segments before the search, so the distribution of the workload between nodes is not as well balanced as for QS-search. The percent load imbalance for DS-BLAST (the time difference between completion of the first and last processors) has been as much as 5% of the total search time; for QS-search, on the other hand, the percent load imbalance is generally much less than 2%, and approached 3% only when 64 processors were used (data not shown). The merge phase of QS-search consists entirely of concatenating the results provided by each node into a single file. In contrast, the merge phase of DS-BLAST must parse the output from each node and combine the results for each single query sequence. As the number of nodes employed increases, the time required for the merge also increases. The advantage of the database splitting approach under limited memory conditions was not apparent in these benchmarks, since the memory available for each node in the cluster used in these experiments was large enough to accommodate the entire 733 MB database.

We also compared the performance of DS-BLAST to that of mpiBLAST [11] version 1.1.0. We found that DS-BLAST was almost twice as fast as mpiBLAST when both utilized 4 processors and more than twice as fast when both utilized 32 processors (data not shown). Both mpiBLAST and DS-BLAST required a substantial part of the total run time to merge and format the final BLAST output.

## Conclusions

To increase the speed and efficiency of sequence similarity search programs, we have developed the SS-Wrapper package, a series of wrapper applications that supports the deployment of sequence similarity searches on high-performance computing clusters. QS-search implements a query sequence splitting approach for the deployment of NCBI BLAST and HMMPFAM. It also will support other similarity search programs, including all variants of NCBI BLAST (blastn, blastp, blastx, tblastn, and tblastx) as well as all options provided by the blastall executable. Because this implementation does not alter the original program, program updates and new programs should be easily accommodated. The output from QS-search is effectively identical to that produced by the underlying program. QS-search is designed to provide optimal load balancing and maximize resource usage when using computer clusters. The performance gain approaches linearity in proportion to the number of processors employed.

When the database is too large to fit into the physical memory of a single node in the cluster, a database splitting approach should outperform the query splitting approach used by QS-search [11,14]. Therefore as a complementary application, the SS-Wrapper package also includes DS-BLAST, which implements a database splitting approach for BLAST searches and provides an effective solution to recalculate the E-value during the post-search phase of processing.

SS-Wrapper provides a suite of tools that makes large sequence similarity searches feasible by deploying the search on a Linux cluster. These tools permit the bioinformatics community to take advantage of the power of high-performance cluster computing. Other tools such as Disperse [20] and TurboBLAST [21] are designed to deploy bioinformatics applications onto loosely connected machines. A more general approach to deployment uses grid computing as an increasingly popular alternative to cluster computing [22]. Grid computing organizes widespread, diverse collections of CPU resources (including desktop workstations, servers, and clusters) into a virtual supercomputer, where these collections of hardware, software, and data resources are organized into a more uniform, manageable, visual whole. In contrast, the CPUs in a Linux cluster are more tightly coupled and specialized. Grid computing has the advantage of utilizing large numbers of CPUs as they become available to the grid. The disadvantage of a grid is in managing the complexity of the disparate architectures of the available CPUs, minimizing overhead, and making maximum usage of network bandwidth. For maximal performance, tools in the SS-Wrapper package have been developed under the assumption of a homogenous Linux cluster in which every CPU is similar. We are currently exploring methods to extend our current work to take advantage of grid computing technologies. To accomplish this, the complexities involved will require significant modification and extension of the applications that are a part of the SS-Wrapper package.

## Availability and requirements

The SS-Wrapper package is freely available under the Artistic License described in the Open Source Initiative. The source code can be downloaded via ftp [17]. Contact elliotl@uab.edu for information on obtaining the software. All tools have been tested on an IBM Intel^® ^processor-based Linux cluster with LAM/MPI [23] and should be compatible with other implementations of MPI.

## Authors' contributions

CW was responsible for the conception, design, implementation, and testing of the SS-Wrapper package. EJL contributed to its conception and testing and provided overall project coordination. Both authors have read and approved the final manuscript.
